# The association between perceived stress and career adaptability: the mediating role of meaning in life and the moderating role of future work self salience

**DOI:** 10.3389/fpsyg.2026.1891424

**Published:** 2026-10-05

**Authors:** Wen Hu, Wenbo Chen, Hewen Wu

**Affiliations:** 1 School of Teacher Development, Shaanxi Normal University, Xi’an, China; 2 Student Affairs Department, Xihang University, Xi’an, China

**Keywords:** career adaptability, college students, future work self salience, meaning in life, moderated mediation, perceived stress

## Abstract

**Introduction:**

Career adaptability is an important psychosocial resource that helps college students prepare for academic demands, employment uncertainty, and the transition from university to work. However, less is known about how perceived stress is associated with career adaptability in the context of the university-to-work transition. Guided by Conservation of Resources theory, this study examined whether meaning in life statistically accounts for part of the association between perceived stress and career adaptability, and whether future work self salience moderates the association between perceived stress and meaning in life.

**Methods:**

In this cross-sectional study, 1,893 students from three universities in Shaanxi, Sichuan, and Qinghai completed self-report measures of perceived stress, meaning in life, future work self salience, and career adaptability. Descriptive statistics, Pearson correlations, and regression-based moderated mediation analyses were conducted.

**Results:**

Perceived stress was negatively associated with career adaptability. Meaning in life statistically accounted for part of this association, with higher perceived stress associated with lower meaning in life and lower meaning in life associated with lower career adaptability in the tested model. Future work self salience moderated the association between perceived stress and meaning in life. Specifically, the negative association between perceived stress and meaning in life was weaker among students with higher future work self salience. The conditional indirect association between perceived stress and career adaptability through meaning in life was smaller in absolute magnitude at higher levels of future work self salience.

**Discussion:**

The findings in a three-university sample suggest a moderated mediation pattern in which meaning in life statistically accounts for part of the association between perceived stress and career adaptability, and future work self salience moderates the association between perceived stress and meaning in life.

## 1 Introduction

Career adaptability refers to the psychosocial resources that individuals draw on to manage vocational tasks, transitions, and work-related difficulties. Career construction theory defines career adaptability as readiness to respond to developmental and transitional demands in the career domain. Its four dimensions, namely concern, control, curiosity and confidence, reflect the students’ attitude towards the future, the responsibility for their career choices, the exploration of occupational opportunities and the confidence in dealing with career problems (Savickas and Porfeli, 2012; Johnston, 2018). These resources are particularly important during the university- to-work transition, when students have to make career decisions under academic stress and unclear labour market conditions (Rudolph et al., 2017). Recent longitudinal studies indicate that career adaptability grows along with academic self-efficacy throughout the college period, suggesting that it should be regarded as a changeable developmental resource rather than a stable trait (Yi et al., 2025). Furthermore, intervention studies demonstrate that career adaptability can be improved by means of specific training and structured development programmes, thus making it an important topic in university career education (Koen et al., 2012; Wang et al., 2024).

Among university students, career adaptability is related to transition preparation and psychological adjustment. Those who have higher adaptability usually participate more in career exploration, job-preparation and career decision-making, and show better ability to keep their adjustment under uncertain employment conditions (Hou et al., 2012; Johnston, 2018). Research evidence from Chinese higher education indicates the relationship between career adaptability and mental health, job-search behavior, subjective happiness and future-oriented career regulation (Gao et al., 2025; Ouyang et al., 2025). Recent longitudinal research has reported associations among resilience, career decision-making self-efficacy, and career adaptability, providing temporal evidence for relationships among these personal resources (Alkal, 2025). Career education skills are connected with career adaptability via career decision-making self-efficacy (Liu et al., 2023), and recent investigations focus on teacher support and future work self salience as contextual and self-regulating resources associated with career adaptability among college students (Zhang et al., 2025b). These results connect career adaptability with the aspects of career preparation, self-regulation and psychological functioning.

Western China provides a meaningful context for examining these issues. In November 2024, China’s Ministry of Education projected that the number of college graduates in 2025 would reach 12.22 million (Ministry of Education of the People’s Republic of China, 2024). Research on Chinese college students’ employment anxiety has identified concerns about personal ability, knowledge application, career replaceability, and social relations (Wang C. et al., 2025). Perceived academic stress has also been positively associated with employment anxiety, with psychological resilience statistically accounting for part of this association (Yang et al., 2025). These findings highlight the relevance of considering students’ psychological resources alongside their academic and employment-related concerns. Against this background, the present study examines the association between perceived stress and career adaptability among students at three universities in western China. Drawing on Conservation of Resources theory, we test whether meaning in life statistically accounts for part of this association and whether future work self salience moderates the association between perceived stress and meaning in life.

## 2 Literature review and research hypotheses

### 2.1 Perceived stress and career adaptability

Conservation of Resources (COR) theory is the basis for the proposed model. Perceived stress is defined as the judgment that the demands of a situation are greater than the available coping resources, not merely due to the presence of objective demands (Cohen et al., 1983; Cohen et al., 2007). COR theory suggests that people try to obtain, maintain and preserve valuable resources, and stress occurs when these resources are endangered, lost or not adequately recovered after being invested in (Hobfoll, 1989; Hobfoll et al., 2018). These resources comprise both external circumstances and personal, psychological and motivational advantages which aid in achieving goals (Halbesleben et al., 2014). For college students, higher perceived stress may be associated with fewer resources available for activities such as career planning, occupational exploration, independent decision-making and building confidence.

Empirical evidence is consistent with this resource-based explanation. Prior research has shown that perceived stress is negatively associated with career adaptability among university students, especially under conditions of uncertainty such as the COVID-19 pandemic (Zhuang et al., 2021). Related studies also suggest that students’ adaptive career resources are closely connected with career decision-making, job-search preparation, and psychological adjustment during the school-to-work transition (Chen et al., 2022). Moreover, career adaptability has been associated with future time perspective and career anxiety among undergraduates, indicating that students’ adaptive career resources are intertwined with how they anticipate and cope with future career uncertainty (Boo et al., 2021). These findings provide a basis for expecting a negative association between perceived stress and career adaptability.

*Hypothesis 1*: Perceived stress is negatively associated with career adaptability.

### 2.2 The mediating role of meaning in life

Meaning in life refers to the sense that one’s life is coherent, purposeful, and significant (Steger, 2012; Martela and Steger, 2016). Theoretical accounts emphasize its relevance to organizing goals, sustaining effort and interpreting stressful situations within a broader life direction (King and Hicks, 2021; Park, 2010). For college students, meaning in life may be especially important during the university-to-work transition, given its proposed role in connecting current academic effort with future personal and occupational goals.

From a COR perspective, a negative association between perceived stress and meaning in life would be consistent with the proposed depletion of resources needed to maintain purpose, coherence, and future direction. Students facing academic and employment-related pressures may perceive their current efforts as fragmented or disconnected from their long-term career goals. In this sense, meaning in life can be viewed as a resource that helps students interpret present demands within a coherent developmental trajectory. Consistent with this view, research with Chinese college students has shown that future-oriented cognition and resilience are associated with both perceived stress and meaning in life, highlighting the relevance of meaning-related resources in stress regulation (Wang N. et al., 2025). Daily-life and coping research has also linked meaning in life with adjustment to daily stressors, positive affect and proactive coping (Park and Baumeister, 2017; Miao et al., 2017).

Lower meaning in life may also be associated with lower career adaptability. Career adaptability requires concern for the future, control over career decisions, curiosity about occupational possibilities, and confidence in coping with career-related challenges. These adaptive responses may be less evident when students lack a sense of purpose or perceive their current efforts as disconnected from future goals. In the career domain, purpose and meaning have been treated as motivational resources that support vocational development and persistence (Dik et al., 2015). Prior research has also linked life meaning with future-oriented career development and calling (Zhang et al., 2017), and recent evidence has reported a positive association between meaning in life and career adaptability among college students (Lin, 2024). Therefore, meaning in life may statistically account for part of the association between perceived stress and career adaptability.

*Hypothesis 2*: Meaning in life statistically accounts for part of the association between perceived stress and career adaptability.

### 2.3 Future work self salience as a moderator

Students reporting higher stress may differ in their levels of meaning in life. Some report a clear sense of direction under pressure, whereas others report less purpose and coherence. Future work self salience may moderate the association between perceived stress and meaning in life. The construct refers to the clarity and accessibility of one’s imagined future occupational self (Strauss et al., 2012). The possible selves perspective suggests that individuals are guided by cognitive representations of what they might become, would like to become, or are afraid of becoming in the future (Markus and Nurius, 1986). Building on this perspective, future work self salience describes the extent to which a desired future occupational self is clear, accessible, and motivationally relevant (Talluri et al., 2025). A salient future work self may therefore provide students with a concrete image of the person they hope to become in the work domain and direct attention and effort toward long-term career goals (Anseel et al., 2018).

Prior research has linked future work self salience to proactive career behavior, consistent with its conceptualization as a future-oriented motivational resource (Taber and Blankemeyer, 2015). Studies of new labor-market entrants and job seekers have further shown that future work self salience is associated with career adaptability, career exploration, job-search behavior, and job-search outcomes, suggesting its relevance to preparation for the school-to-work transition (Guan et al., 2014; Fang and Saks, 2022). Evidence from Chinese college students also connects future work self salience with career adaptability and future-oriented career regulation, showing its relevance in higher education contexts (Ling et al., 2022; Lu and Jia, 2022). More recent studies have linked future work self salience with employability through career exploration and with lower career distress through goal engagement (Ma et al., 2024; Xu and Yu, 2025). These findings provide a basis for examining future work self salience as a potential moderator of the association between perceived stress and meaning in life.

Within COR theory, future work self salience can be viewed as a future-oriented personal resource in the stress-meaning pathway. A salient future occupational self may help students interpret academic and employment-related pressure as part of an ongoing career-development process, rather than as a disconnected threat. Such an interpretation may be associated with a stronger sense of vocational purpose and meaning in life under pressure. Students with a less salient future work self may have fewer cognitive anchors for interpreting current strain. Accordingly, the negative association between perceived stress and meaning in life is expected to be weaker at higher levels of future work self salience. If meaning in life statistically accounts for part of the stress–adaptability association, this moderation should also be reflected in the size of the conditional indirect association.

*Hypothesis 3*: Future work self salience moderates the association between perceived stress and meaning in life, such that this negative association is weaker among students with higher future work self salience.

The present study tests a cross-sectional moderated mediation model among students at three universities in western China. The model examines the association between perceived stress and career adaptability, the mediating role of meaning in life, and the moderating role of future work self salience in the association between perceived stress and meaning in life. The proposed model is shown in Figure 1.

**Figure 1:**
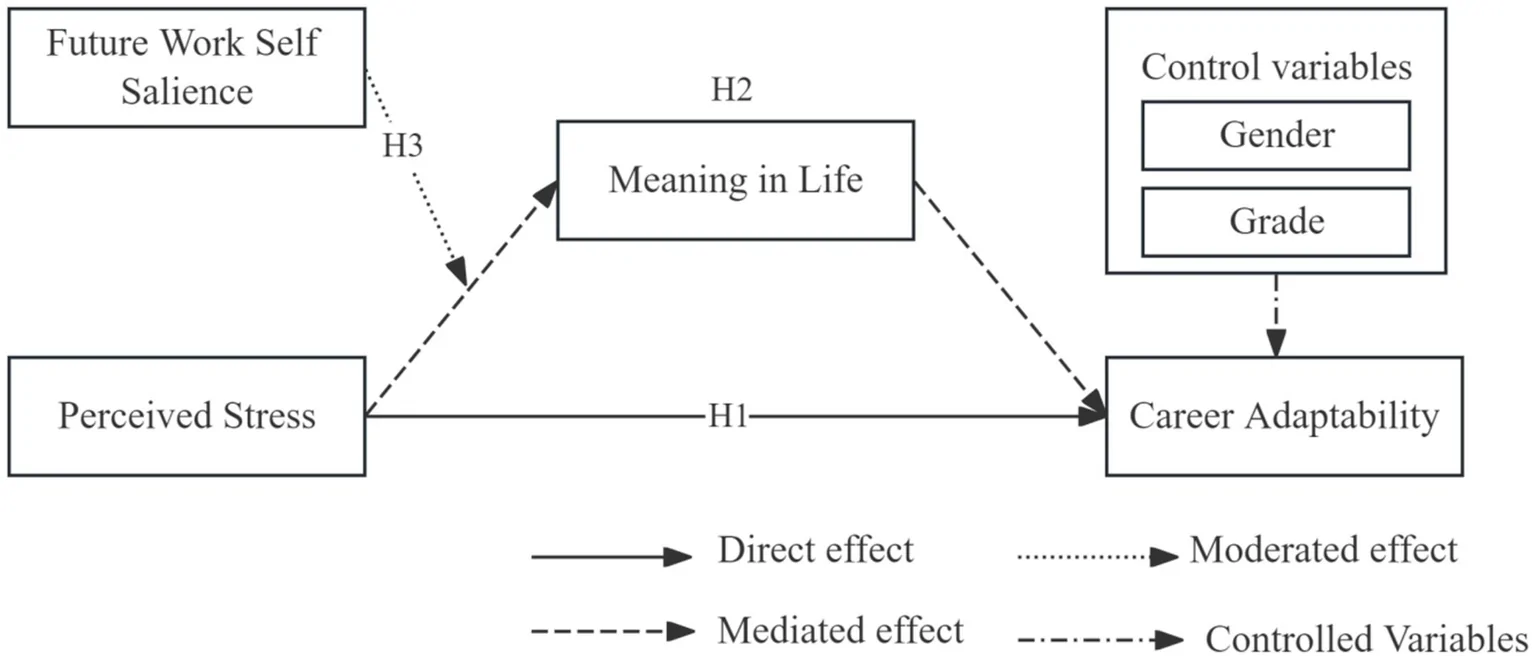
The conceptual framework of this research.

## 3 Materials and methods

### 3.1 Participants

This cross-sectional survey was conducted between April 20 and May 1, 2025, at three universities in western China: Xihang University in Shaanxi, Chengdu Technological University in Sichuan, and Qinghai Minzu University in Qinghai. Participants were recruited through stratified convenience sampling, with grade as the stratification variable and students from all four undergraduate years invited. Eligible participants were full-time undergraduates aged 18 years or older enrolled at one of the participating universities.

A total of 2,170 students were invited, and 1,967 submitted questionnaires, yielding a response rate of 90.6%. Responses were screened for completion times of less than 120 s, straight-lining, and missing or invalid data. After excluding 74 responses that met one or more of these criteria, 1,893 were retained for analysis, representing 96.2% of submitted questionnaires.

The final sample included 1,027 male students (54.3%) and 866 female students (45.7%). By grade, 559 were freshmen (29.5%), 514 sophomores (27.2%), 465 juniors (24.6%), and 355 seniors (18.8%). Of the participants, 669 (35.3%), 578 (30.5%), and 646 (34.1%) were enrolled at the universities in Shaanxi, Sichuan, and Qinghai, respectively.

### 3.2 Procedure

An anonymous Chinese-language questionnaire was administered through Questionnaire Star. University staff or student representatives distributed the survey link through university instructional systems and student WeChat groups. Each student was permitted to submit only one questionnaire. Before responding, participants read an electronic consent form explaining the study purpose, voluntary participation, confidentiality, data security, and the right to withdraw. Only those who provided electronic informed consent could proceed.

### 3.3 Measurement of variables

For all multi-item measures, scores were calculated by averaging item scores after reverse scoring where applicable. The response scales and scoring procedures used in this study, together with the Chinese items and English reporting translations, are provided in Supplementary material 2.

#### 3.3.1 Perceived stress

Perceived stress was assessed using the Chinese version of the Perceived Stress Scale (PSS; Cohen et al., 1983; Yang and Huang, 2003). This 14-item scale evaluates the sense of tension and lack of ability to control. For instance, the questions are “During the last month, how often did you feel nervous and stressed?” and “During the last month, how often did you think that you could not handle the important matters in your life?” The scores of the items range from 1 (never) to 5 (always), and some items are inverted. After reverse scoring the relevant items, item scores were averaged, with higher mean scores indicating greater perceived stress. In the present study, Cronbach’s alpha was 0.892 for the total scale, 0.867 for tension, and 0.898 for lack of control. Confirmatory factor analysis (CFA) of the PSS measurement model yielded *χ*^2^/*df* = 4.446 (*χ*^2^ = 333.426, *df* = 75), CFI = 0.979, TLI = 0.974, RMSEA = 0.043, and SRMR = 0.031.

#### 3.3.2 Career adaptability

Career adaptability was evaluated using the Chinese version of the Career Adapt-Abilities Scale (CAAS), which was established by Savickas and Porfeli and confirmed among Chinese university students by Hou et al. (Savickas and Porfeli, 2012; Hou et al., 2012). This scale contains 24 items and measures concern, control, curiosity and confidence. Some examples of the items are “Considering what my future will be like,” “Being responsible for my own actions,” “Exploring my environment” and “Completing tasks effectively.” Responses ranged from 1 (does not describe me at all) to 5 (describes me completely). Item scores were averaged, with higher mean scores indicating greater career adaptability. Cronbach’s alpha was 0.948 for the total scale, with subscale values of 0.889 for concern, 0.891 for control, 0.901 for curiosity and 0.869 for confidence. CFA of the correlated four-factor model yielded *χ*^2^/*df* = 1.487 (*χ*^2^ = 365.701, *df* = 246), CFI = 0.995, TLI = 0.995, RMSEA = 0.016, and SRMR = 0.014.

#### 3.3.3 Meaning in life

Meaning in life was evaluated using the Chinese version of the Meaning in Life Questionnaire (MLQ), which was established by Steger et al. and verified in Chinese populations by Liu and Gan (Steger et al., 2006; Liu and Gan, 2010). This 10-item scale contains the aspects of presence of meaning and search for meaning. Some examples of items are “I know the meaning of my life” and “I am seeking something that gives my life significance.” The items were rated from 1 (completely disagree) to 7 (completely agree). Item 9 of the MLQ was reverse-scored before calculating the composite and subscale scores. The mean of all 10 items was used as the composite meaning-in-life score in the primary analyses. For the sensitivity analyses (Section 4.5), separate five-item mean scores were calculated for presence of meaning (items 1, 4, 5, 6, and 9) and search for meaning (items 2, 3, 7, 8, and 10). Cronbach’s alpha was 0.891 for the total scale, 0.878 for presence of meaning, and 0.871 for search for meaning. CFA of the correlated two-factor model distinguishing presence of meaning and search for meaning yielded *χ*^2^/*df* = 1.528 (*χ*^2^ = 51.936, *df* = 34), CFI = 0.998, TLI = 0.997, RMSEA = 0.017, and SRMR = 0.015.

#### 3.3.4 Future work self salience

Future work self salience (FWSS) was measured using the five-item scale developed to assess how clearly and vividly individuals can imagine their future work self (Strauss et al., 2012). Sample items include “I can easily imagine my future work self,” “The mental picture of this future is very clear,” and “I am very clear about who and what I want to be in my future work.” The scale was translated into Chinese and then back-translated by two bilingual researchers. Discrepancies were resolved through discussion to ensure semantic equivalence and cultural appropriateness. Responses ranged from 1 (does not describe me at all) to 5 (describes me completely). Item scores were averaged, with higher mean scores indicating greater future work self salience. Cronbach’s alpha was 0.894 in the present study. CFA of the measurement model yielded *χ*^2^/*df* = 4.818 (*χ*^2^ = 19.271, *df* = 4), CFI = 0.997, TLI = 0.993, RMSEA = 0.045, and SRMR = 0.010.

### 3.4 Data processing and statistical analysis

Analyses were performed in SPSS 27.0 with PROCESS macro version 4.1. Data were screened for missing values, invalid responses, and abnormal response patterns before analysis. Descriptive statistics and Pearson correlations were calculated for all study variables, and internal consistency was evaluated with Cronbach’s alpha.

Potential common method bias was assessed using Harman’s single-factor test and an unmeasured latent method factor (ULMC) analysis (Podsakoff et al., 2012). The ULMC analysis compared the baseline measurement model with a model including an additional method factor. CFA and the ULMC analysis were conducted using IBM SPSS Amos 31. Model fit was evaluated using *χ*^2^/*df*, CFI, TLI, RMSEA, and SRMR. The statistical indirect association between perceived stress and career adaptability through meaning in life was tested with PROCESS Model 4. PROCESS Model 7 was then used to test whether future work self salience moderated the association between perceived stress and meaning in life and whether the indirect association varied across levels of the moderator. Gender and grade were included as covariates. The analyses followed the conditional process framework for estimating indirect and conditional indirect effects with bootstrapped confidence intervals (Hayes, 2022).

Continuous variables were mean-centered before the interaction term was entered. Bootstrap confidence intervals were estimated using 5,000 resamples. Effects were considered statistically significant when the 95% confidence interval did not include zero.

## 4 Results

### 4.1 Common method bias test

All variables were measured by self-report, so Harman’s single-factor test was used as an initial diagnostic. The first unrotated factor explained 31.635% of the total variance. This finding did not indicate a dominant single factor but could not rule out common method variance (Podsakoff et al., 2012).

Common method variance was further examined by comparing the baseline measurement model with a model including an unmeasured latent method factor (ULMC). The baseline model yielded *χ*^2^/*df* = 1.423 (*χ*^2^ = 1834.156, *df* = 1,289), CFI = 0.990, TLI = 0.989, RMSEA = 0.015, and SRMR = 0.019. The model including the method factor yielded *χ*^2^/*df* = 1.208 (*χ*^2^ = 1493.419, *df* = 1,236), CFI = 0.995, TLI = 0.995, RMSEA = 0.010, and SRMR = 0.016.

Relative to the baseline model, CFI increased by 0.005 and TLI by 0.006, whereas RMSEA decreased by 0.005 and SRMR by 0.003. These changes were small in absolute terms, but they do not establish the absence of common method bias. The regression estimates should therefore not be interpreted as corrected for method bias.

### 4.2 Descriptive statistics and correlation analysis

Table 1 presents the descriptive statistics and correlations. Perceived stress was negatively correlated with meaning in life (*r* = −0.436, *p* < 0.01), career adaptability (*r* = −0.481, *p* < 0.01), and future work self salience (*r* = −0.478, *p* < 0.01). Meaning in life was positively correlated with career adaptability (*r* = 0.541, *p* < 0.01) and future work self salience (*r* = 0.582, *p* < 0.01). Career adaptability was positively correlated with future work self salience (*r* = 0.532, *p* < 0.01). The correlation pattern was consistent with the proposed model.

**Table 1:** Descriptive statistics and correlations among study variables.

Variable	M	SD	1	2	3	4
1. Perceived stress	2.421	0.702	1			
2. Meaning in life	5.042	1.106	−0.436^**^	1		
3. Career adaptability	3.900	0.656	−0.481^**^	0.541^**^	1	
4. Future work self salience	3.558	0.827	−0.478^**^	0.582^**^	0.532^**^	1

*N* = 1,893. M and SD are based on item-level mean scores. *p < 0.05, **p < 0.01, ***p < 0.001.

### 4.3 The mediating role of meaning in life

Meaning in life was tested as a mediator using PROCESS Model 4, with gender and grade entered as covariates. As shown in Table 2, perceived stress was negatively associated with career adaptability (*b* = −0.450, 95% CI [−0.487, −0.413], *t* = −23.922, *p* < 0.001) and meaning in life (*b* = −0.687, 95% CI [−0.751, −0.623], *t* = −21.071, *p* < 0.001). When perceived stress and meaning in life were entered together, meaning in life was positively associated with career adaptability (*b* = 0.242, 95% CI [0.219, 0.266], *t* = 20.098, *p* < 0.001), and the direct association between perceived stress and career adaptability remained significant (*b* = −0.283, 95% CI [−0.321, −0.246], *t* = −14.943, *p* < 0.001). The bootstrapped indirect effect through meaning in life was significant (effect = −0.167, 95% CI [−0.201, −0.135]), accounting for 37.1% of the total effect. These results support Hypotheses 1 and 2.

**Table 2:** Mediation analysis of meaning in life.

Variables		Overall fit index				95% CI		
Result	Predictor	*R*	*R* ^2^	*F*	*b*	LLCI	ULCI	*t*
Career adaptability	Gender	0.483	0.233	191.185^***^	0.036	−0.016	0.089	1.370
	Grade				−0.012	−0.036	0.012	−0.957
	Perceived stress				−0.450	−0.487	−0.413	−23.922^***^
Meaning in life	Gender	0.437	0.190	148.378^***^	−0.000	−0.090	0.090	−0.002
	Grade				−0.025	−0.066	0.017	−1.170
	Perceived stress				−0.687	−0.751	−0.623	−21.071^***^
Career adaptability	Gender	0.606	0.368	274.950^***^	0.036	−0.011	0.084	1.510
	Grade				−0.005	−0.027	0.016	−0.513
	Perceived stress				−0.283	−0.321	−0.246	−14.943^***^
	Meaning in life				0.242	0.219	0.266	20.098^***^

*N* = 1,893, *p < 0.05, **p < 0.01, ***p < 0.001.

### 4.4 The moderating role of future work self salience

PROCESS Model 7 was used to examine whether future work self salience moderated the association between perceived stress and meaning in life, after controlling for gender and grade. As shown in Table 3, the interaction between perceived stress and future work self salience was significant (*b* = 0.157, 95% CI [0.100, 0.214], *t* = 5.379, *p* < 0.001), indicating that the stress–meaning association varied by the level of future work self salience. Simple slope analyses showed that perceived stress was more strongly and negatively associated with meaning in life at low levels of future work self salience (b = −0.450, *t* = −11.203, *p* < 0.001) than at high levels of future work self salience (*b* = −0.190, *t* = −4.654, *p* < 0.001). The simple-slope pattern is shown in Figure 2. These findings were consistent with Hypothesis 3.

**Table 3:** Moderated mediation model with future work self salience as a moderator.

Variables		Overall fit index				95% CI		
Result	Predictor	*R*	*R* ^2^	*F*	*b*	LLCI	ULCI	*t*
Meaning in life	Gender	0.617	0.380	231.422^***^	−0.009	−0.088	0.071	−0.211
	Grade				−0.009	−0.045	0.027	−0.486
	Perceived stress				−0.320	−0.384	−0.256	−9.834^***^
	Future work self salience				0.631	0.577	0.686	22.742^***^
	Perceived stress × Future work self salience				0.157	0.100	0.214	5.379^***^
Career adaptability	Gender	0.607	0.368	274.950^***^	0.036	−0.011	0.084	1.510
	Grade				−0.006	−0.027	0.016	−0.513
	Perceived stress				−0.284	−0.321	−0.247	−14.943^***^
	Meaning in life				0.242	0.219	0.266	20.098^***^

*N* = 1,893, *p < 0.05, **p < 0.01, ***p < 0.001.

**Figure 2:**
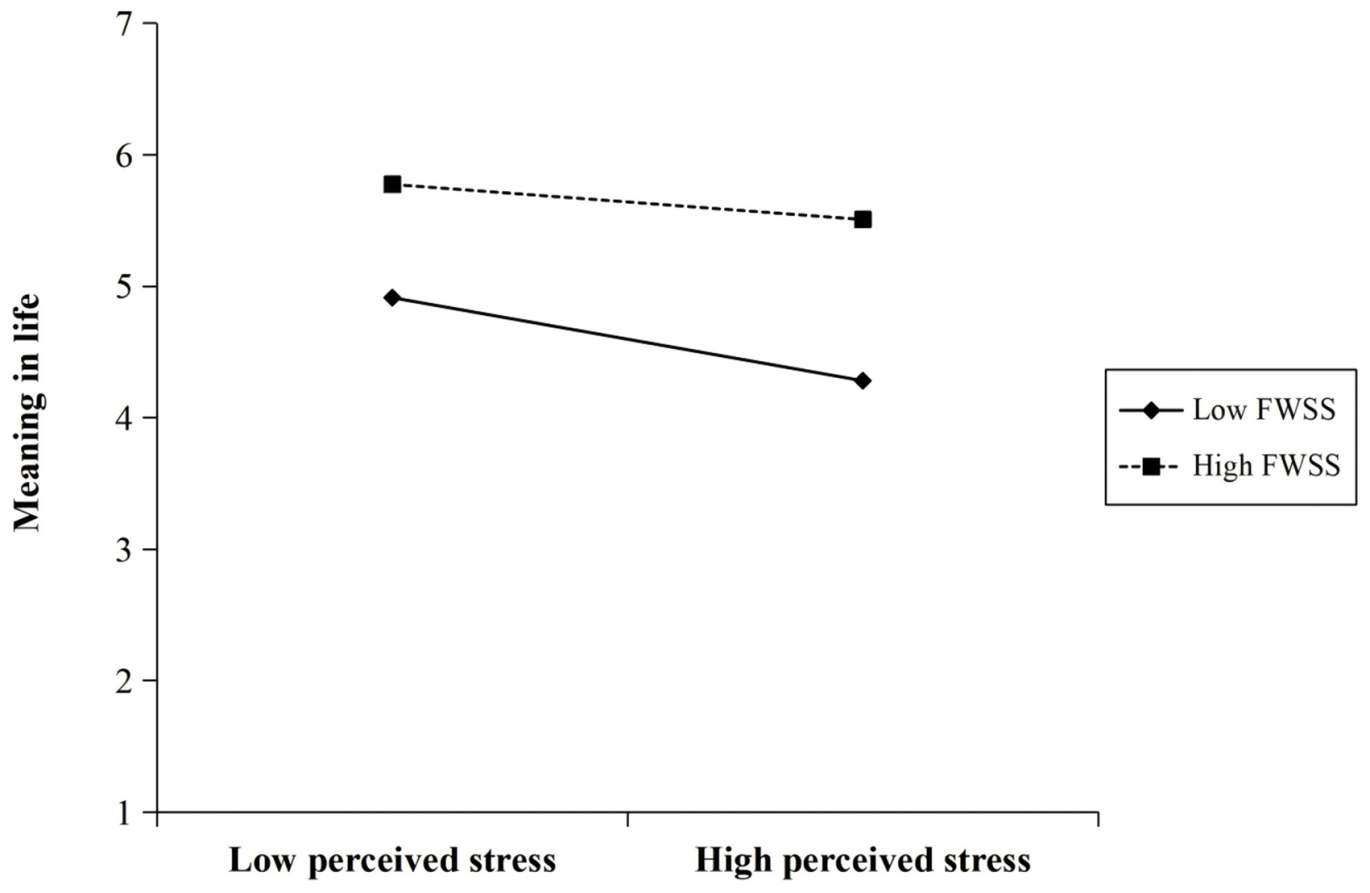
Association between perceived stress and meaning in life across levels of future work self salience.

Conditional indirect effects were estimated at low, mean, and high levels of future work self salience (Table 4). The conditional indirect association between perceived stress and career adaptability through meaning in life was significant at low (effect = −0.109, Boot SE = 0.016, 95% CI [−0.142, −0.080]), mean (effect = −0.078, Boot SE = 0.010, 95% CI [−0.099, −0.059]), and high levels of future work self salience (effect = −0.046, Boot SE = 0.010, 95% CI [−0.067, −0.026]). The conditional indirect association was smaller in absolute magnitude at higher levels of future work self salience. The index of moderated mediation was significant (index = 0.038, Boot SE = 0.010, 95% CI [0.019, 0.059]), indicating that the indirect effect varied by future work self salience.

**Table 4:** Conditional indirect effects and index of moderated mediation.

Moderator	Effect	Boot SE	95% CI	
			LLCI	ULCI
M-1SD	−0.109	0.016	−0.142	−0.080
M	−0.078	0.010	−0.099	−0.059
M + 1SD	−0.046	0.010	−0.067	−0.026
Index of moderated mediation	0.038	0.010	0.019	0.059

Boot SE = bootstrap standard error; CI = confidence interval based on 5,000 bootstrap samples. Conditional indirect effects are considered significant when the 95% CI does not include zero.

### 4.5 Sensitivity analyses

To examine whether the findings depended on the composite meaning-in-life score, we repeated PROCESS Models 4 and 7 with presence of meaning and search for meaning entered separately as the mediator. All analyses used the same sample of 1,893 participants, controlled for gender and grade, and used 5,000 bootstrap resamples.

As shown in Supplementary Table S1, the perceived stress × FWSS interaction was positive and significant for both presence of meaning (*b* = 0.1244, *p* = 0.0004) and search for meaning (*b* = 0.1898, *p* < 0.001). Supplementary Table S2 shows that both dimensions had significant negative indirect associations in Model 4. The indices of moderated mediation in Model 7 were also significant for presence of meaning (index = 0.0218, 95% bootstrap CI [0.0067, 0.0382]) and search for meaning (index = 0.0328, 95% bootstrap CI [0.0157, 0.0517]).

The conditional indirect association through presence of meaning was significant at low, mean, and high FWSS levels. For search for meaning, it was significant at low and mean levels, but its confidence interval included zero at high FWSS (estimate = −0.0190, 95% bootstrap CI [−0.0375, 0.0002]). Overall, the sensitivity analyses provided support for the robustness of the general moderated mediation pattern when the two meaning dimensions were examined separately, although the conditional indirect association through search for meaning was not statistically distinguishable from zero at high FWSS. These separate models did not directly test differences between the two dimensions.

## 5 Discussion

This study examined the associations among perceived stress, meaning in life, future work self salience, and career adaptability in a sample of students from three universities in western China. Three findings emerged. Perceived stress was negatively associated with career adaptability. Meaning in life statistically accounted for part of this association. Future work self salience moderated the stress–meaning association, and the conditional indirect association through meaning in life was smaller in absolute magnitude at higher levels of future work self salience. Together, these findings highlight the relevance of meaning in life and future work self salience to the association between perceived stress and career adaptability.

### 5.1 The relationship between perceived stress and career adaptability

Perceived stress was negatively associated with career adaptability, supporting Hypothesis 1. Students who reported greater stress also reported fewer adaptive career resources. From a COR perspective, this association may reflect the proposed tension between resources devoted to immediate demands and those available for long-term career planning, occupational exploration, and confidence-building. This is a theoretical interpretation; the study did not directly measure resource allocation or changes in these resources over time.

This finding is consistent with prior evidence showing a negative association between perceived stress and career adaptability among university students during periods of uncertainty, such as the COVID-19 pandemic (Zhuang et al., 2021). It also extends previous research by treating perceived stress not only as a general psychological burden but also as a condition under which students may report fewer adaptive career resources. Previous studies have shown that career adaptability is closely related to career decision-making, job-search behavior, subjective well-being, and career anxiety (Boo et al., 2021; Chen et al., 2022; Ouyang et al., 2025). These findings highlight the relevance of career adaptability to the school-to-work transition. The present study adds to this literature by showing that perceived stress is negatively associated with this resource among college students, complementing recent evidence linking career adaptability with occupational stress and decent work outcomes in early career populations (Marcionetti et al., 2025).

The present study adds evidence from students at three universities in Shaanxi, Sichuan, and Qinghai, highlighting the relevance of perceived stress to career adaptability in this educational context. The negative association observed in this sample suggests that students’ stress experiences warrant attention alongside their resources for career preparation. Accordingly, career guidance and student support at the participating universities may consider perceived stress alongside occupational goals, career exploration, and decision-making needs.

### 5.2 The mediating role of meaning in life

Meaning in life statistically accounted for part of the negative association between perceived stress and career adaptability, which was consistent with Hypothesis 2. Higher perceived stress was associated with lower meaning in life, and lower meaning in life was associated with lower career adaptability in the tested model. From a resource-based perspective, this pattern may be understood in terms of the connection between present academic effort and broader personal and occupational goals. A weaker sense of this connection may coincide with fewer perceived resources for career planning and exploration.

Related evidence shows that perceived stress, affect, and meaning in life co-fluctuate within persons over time (Moss et al., 2023). Research with Chinese young adults also suggests that perceived stress is closely linked to meaning-related well-being processes, with hope also implicated in these associations (Zhang et al., 2025a). In addition, evidence from Chinese college students has linked presence of meaning in life with perceived stress and adjustment outcomes, highlighting the relevance of meaning-related resources to stress-related adjustment (Huang et al., 2024). These findings support the relevance of meaning in life to stress-related adjustment and provide a basis for examining the indirect association specified in the present model.

The finding also adds evidence regarding the association between meaning in life and career adaptability. Career adaptability encompasses concern for the future, control over career decisions, curiosity about occupational possibilities, and confidence in addressing career challenges. These resources may be related to students’ perceptions that their current efforts are purposeful and connected to future goals. Prior research has linked life meaning with future-oriented career development and calling (Zhang et al., 2017), and recent evidence has reported a positive association between meaning in life and career adaptability among college students (Lin, 2024). Related evidence from vocational students further suggests that associations of motivational and self-regulatory factors with career adaptability may differ across levels of meaning in life (Chuang et al., 2022). The present finding extends this literature to the indirect association between perceived stress and career adaptability through meaning in life.

The dimension-specific sensitivity analyses provided further support for the mediation pattern: both presence of meaning and search for meaning showed significant negative indirect associations when examined separately in Model 4. These findings highlight the relevance of considering both dimensions in the stress–adaptability association. Presence of meaning concerns the experience that life is meaningful, whereas search for meaning concerns efforts to find or deepen meaning (Steger et al., 2006). Thus, the two dimensions showed a similar direction of association while representing distinct aspects of meaning in life.

In the primary model, the direct association between perceived stress and career adaptability remained significant after including meaning in life, a pattern consistent with partial statistical mediation. Resilience, career decision self-efficacy, hope, future time perspective, and perceived career competence may also be relevant to the association between perceived stress and career adaptability. For example, perceived academic stress has shown a statistical indirect association with employment anxiety through psychological resilience among college students (Yang et al., 2025). Future studies could test meaning in life alongside these resources to examine how different psychological resources relate to career adaptability in the context of stress.

### 5.3 The moderating role of future work self salience

Future work self salience moderated the association between perceived stress and meaning in life, which was consistent with Hypothesis 3. The negative association between perceived stress and meaning in life was weaker among students with a more salient future work self. The conditional indirect effects showed a similar pattern: the conditional indirect association between perceived stress and career adaptability through meaning in life was smaller in absolute magnitude at higher levels of future work self salience. These findings suggest that a clear image of one’s future occupational self may function as a boundary condition in the observed associations among perceived stress, meaning in life, and career adaptability.

The observed moderation is consistent with a self-regulatory interpretation of future work self salience. Students who can picture their future occupational self may interpret current academic and employment-related pressure in relation to longer-term career goals. A clearer future occupational self may thus serve as a cognitive reference point for understanding current demands within a valued career trajectory. This interpretation fits evidence from Chinese college students linking future work self salience and future time perspective with career adaptability (Ling et al., 2022). Related longitudinal evidence also highlights the relevance of future work self salience to goal-directed career regulation, with goal engagement mediating its association with lower career distress among college students (Xu and Yu, 2025).

The dimension-specific sensitivity analyses supported the general moderated mediation pattern, with significant indices of moderated mediation for both presence of meaning and search for meaning. The conditional indirect association through presence of meaning was significant at low, mean, and high levels of future work self salience. For search for meaning, it was significant at low and mean levels, but its confidence interval included zero at the high level. Thus, the overall direction of the findings was consistent across dimensions, while the evidence for a nonzero conditional indirect association was not uniform across all examined levels.

This finding complements previous work linking future work self salience to career behavior. Recent review evidence calls for more research on the mechanisms and boundary conditions through which future work self salience operates (Talluri et al., 2025). The present study responds to this call by examining future work self salience as a moderator of the stress–meaning association, rather than considering it only as a correlate of career outcomes.

### 5.4 Theoretical and practical implications

This study contributes to the career adaptability literature by locating perceived stress within a model of adaptive career resources. Whereas previous studies have often examined career adaptability as a predictor of educational, psychological, and career-related outcomes, the present study focuses on perceived stress as a condition under which students may report fewer adaptive career resources. The findings further suggest that meaning in life statistically accounts for part of the association between perceived stress and career adaptability. In addition, the salience of the future work self was identified as a moderator of the association between perceived stress and meaning in life, thereby connecting COR theory with the possible selves perspective in the context of career development under stress.

The findings also have practical implications for university career guidance and counseling. For students experiencing high levels of stress, career support may extend beyond providing occupational information and job-search skills. Career education may incorporate stress management, meaning-centered reflection, and future work self clarification. These components could provide opportunities for students to discuss their stress experiences, reflect on personal sources of meaning, and clarify their future occupational goals. Previous studies have evaluated brief training, online or group career counseling, and structured career interventions, with findings varying across outcomes (Wetstone and Rice, 2023; Carvalho et al., 2023; Van Der Horst et al., 2021).

In addition, career support may benefit from considering students’ broader relational and educational contexts. For example, research with high school students has linked career-related parental support with learning engagement through dimensions of career adaptability and life meaning, highlighting the relevance of supportive relationships alongside individual psychological resources (Xiao et al., 2024). Practical activities may include reflective writing, career story exercises, mentoring, structured career exploration, interviews with career role models, and reflection on internship experiences. At the participating universities, such activities could be tailored to students’ needs and available career-support resources, and evaluated in future intervention studies.

## 6 Strengths, limitations, and future directions

The study drew on a sample of 1,893 students from three universities and included dimension-specific sensitivity analyses of the proposed moderated mediation model. Several limitations should be considered.

The observed relationships should be interpreted as statistical associations rather than causal effects. The cross-sectional design cannot establish the temporal ordering of perceived stress, meaning in life, future work self salience, and career adaptability. Future longitudinal studies, including cross-lagged analyses, could examine temporal relationships, while experimental studies could test the causal assumptions underlying the proposed model.

The sample was obtained through stratified convenience sampling from three universities in Shaanxi, Sichuan, and Qinghai provinces. The non-probability sampling approach limits the representativeness of the sample, both within the participating institutions and across western China. Replication with more diverse samples and sampling strategies that improve representativeness would help assess generalizability across educational and labour-market contexts.

The study relied on self-report questionnaires, which may be subject to shared method variance and social desirability bias. Although additional common method diagnostics were reported, these findings do not rule out common method bias. Future research could complement self-reports with other-source ratings and behavioral indicators of career exploration.

Although the dimension-specific sensitivity analyses supported the general moderated mediation pattern, the use of composite mean scores in the primary analyses may still obscure dimension-specific conditional associations. The separate analyses controlled for gender and grade but did not directly test differences between the two meaning dimensions in their indirect associations or indices of moderated mediation. Future investigations could directly compare these estimates and examine the four dimensions of career adaptability—concern, control, curiosity, and confidence—to assess whether the observed associations differ across dimensions.

## 7 Conclusion

This study examined the association between perceived stress and career adaptability among students at three universities in western China, with meaning in life as a mediator and future work self salience as a moderator. Perceived stress was negatively associated with career adaptability, and meaning in life statistically accounted for part of this association. The negative association between perceived stress and meaning in life was weaker among students with higher future work self salience, and the conditional indirect association through meaning in life was smaller in absolute magnitude at higher levels of future work self salience.

Sensitivity analyses examining presence of meaning and search for meaning separately supported the general moderated mediation pattern, although the bootstrap confidence interval for the conditional indirect association through search for meaning included zero at high levels of future work self salience. These findings highlight the relevance of meaning in life and the clarity of the future occupational self to the stress–adaptability association in this sample. These cross-sectional associations suggest directions for future evaluation of stress management, meaning-centered reflection, and future work self clarification in career education and counseling.

## Data Availability

The raw data supporting the conclusions of this article will be made available by the corresponding author upon reasonable request.

## Supplementary material

Supplementary Material 1 Statistical results of the dimension-specific sensitivity analyses (Tables S1 and S2), measurement-model fit indices (Table S3), and unmeasured latent method factor model comparison (Table S4).

Supplementary Material 2 Chinese questionnaire items, English reporting translations, response options, and scoring instructions for the perceived stress, career adaptability, meaning in life, and future work self salience measures.
