# Supplementary material for: The association between perceived stress and career adaptability: the mediating role of meaning in life and the moderating role of future work self salience
Source: Front Psychol. 2026 Oct 5;17:1891424. doi: 10.3389/fpsyg.2026.1891424 (PMC13677823; doi:10.3389/fpsyg.2026.1891424)
Supplement: Supplementary file 3 [file Table_1.DOCX]

# Supplementary Material 1 Table S1. Regression results of the dimension-specific analyses.

Presence of meaning and search for meaning were entered separately as mediators in PROCESS Models 4 and 7. All four analyses used N = 1,893 and controlled for gender and grade. The two dimensions were not combined in a parallel-mediator model. Panels A1–B2 report the mediator and outcome equations.

## Panel A1. PROCESS Model 4 — Presence of meaning

### Outcome: Presence of meaning

| **Predictor** | **b** | **SE** | **t** | **p** | **95% CI** |
| --- | --- | --- | --- | --- | --- |
| Intercept | 7.0160 | 0.1331 | 52.6988 | <0.001 | [6.7549, 7.2771] |
| Perceived stress | −0.7508 | 0.0378 | −19.8845 | <0.001 | [−0.8248, −0.6767] |
| Gender | −0.0075 | 0.0533 | −0.1402 | 0.8886 | [−0.1120, 0.0970] |
| Grade | −0.0099 | 0.0244 | −0.4076 | 0.6836 | [−0.0577, 0.0379] |

R² = 0.1732; F(3, 1,889) = 131.9085, p < 0.001.

### Outcome: Career adaptability

| **Predictor** | **b** | **SE** | **t** | **p** | **95% CI** |
| --- | --- | --- | --- | --- | --- |
| Intercept | 3.7355 | 0.0977 | 38.2438 | <0.001 | [3.5439, 3.9271] |
| Perceived stress | −0.3188 | 0.0194 | −16.4476 | <0.001 | [−0.3568, −0.2808] |
| Presence of meaning | 0.1750 | 0.0107 | 16.2969 | <0.001 | [0.1540, 0.1961] |
| Gender | 0.0377 | 0.0249 | 1.5154 | 0.1298 | [−0.0111, 0.0865] |
| Grade | −0.0099 | 0.0114 | −0.8689 | 0.3850 | [−0.0322, 0.0124] |

R² = 0.3275; F(4, 1,888) = 229.8702, p < 0.001.

*Note. b = unstandardized regression coefficient; SE = regression standard error; CI = confidence interval. The confidence intervals in this table are regression intervals, not bootstrap intervals. Indirect estimates are reported in Supplementary Table S2.*

# Supplementary Table S1 (continued).

## Panel A2. PROCESS Model 4 — Search for meaning

### Outcome: Search for meaning

| **Predictor** | **b** | **SE** | **t** | **p** | **95% CI** |
| --- | --- | --- | --- | --- | --- |
| Intercept | 6.5101 | 0.1381 | 47.1273 | <0.001 | [6.2392, 6.7810] |
| Perceived stress | −0.6232 | 0.0392 | −15.9085 | <0.001 | [−0.7001, −0.5464] |
| Gender | 0.0073 | 0.0553 | 0.1318 | 0.8952 | [−0.1011, 0.1157] |
| Grade | −0.0394 | 0.0253 | −1.5560 | 0.1199 | [−0.0890, 0.0103] |

R² = 0.1190; F(3, 1,889) = 85.0242, p < 0.001.

### Outcome: Career adaptability

| **Predictor** | **b** | **SE** | **t** | **p** | **95% CI** |
| --- | --- | --- | --- | --- | --- |
| Intercept | 3.8368 | 0.0913 | 42.0105 | <0.001 | [3.6577, 4.0160] |
| Perceived stress | −0.3423 | 0.0187 | −18.3077 | <0.001 | [−0.3790, −0.3057] |
| Search for meaning | 0.1731 | 0.0103 | 16.7816 | <0.001 | [0.1528, 0.1933] |
| Gender | 0.0351 | 0.0248 | 1.4174 | 0.1565 | [−0.0135, 0.0837] |
| Grade | −0.0048 | 0.0113 | −0.4245 | 0.6712 | [−0.0271, 0.0174] |

R² = 0.3325; F(4, 1,888) = 235.0959, p < 0.001.

*Note. b = unstandardized regression coefficient; SE = regression standard error; CI = confidence interval. The confidence intervals in this table are regression intervals, not bootstrap intervals. Indirect estimates are reported in Supplementary Table S2.*

# Supplementary Table S1 (continued).

## Panel B1. PROCESS Model 7 — Presence of meaning

### Outcome: Presence of meaning

| **Predictor** | **b** | **SE** | **t** | **p** | **95% CI** |
| --- | --- | --- | --- | --- | --- |
| Intercept | 5.2056 | 0.0892 | 58.3507 | <0.001 | [5.0306, 5.3806] |
| Perceived stress | −0.3726 | 0.0389 | −9.5733 | <0.001 | [−0.4489, −0.2962] |
| Future work self salience | 0.6550 | 0.0332 | 19.7181 | <0.001 | [0.5898, 0.7201] |
| Perceived stress × FWSS | 0.1244 | 0.0350 | 3.5596 | 0.0004 | [0.0559, 0.1930] |
| Gender | −0.0142 | 0.0482 | −0.2949 | 0.7681 | [−0.1088, 0.0804] |
| Grade | 0.0061 | 0.0221 | 0.2765 | 0.7822 | [−0.0372, 0.0494] |

R² = 0.3237; F(5, 1,887) = 180.6220, p < 0.001.

### Outcome: Career adaptability

| **Predictor** | **b** | **SE** | **t** | **p** | **95% CI** |
| --- | --- | --- | --- | --- | --- |
| Intercept | 2.9638 | 0.0721 | 41.1154 | <0.001 | [2.8224, 3.1051] |
| Perceived stress | −0.3188 | 0.0194 | −16.4476 | <0.001 | [−0.3568, −0.2808] |
| Presence of meaning | 0.1750 | 0.0107 | 16.2969 | <0.001 | [0.1540, 0.1961] |
| Gender | 0.0377 | 0.0249 | 1.5154 | 0.1298 | [−0.0111, 0.0865] |
| Grade | −0.0099 | 0.0114 | −0.8689 | 0.3850 | [−0.0322, 0.0124] |

R² = 0.3275; F(4, 1,888) = 229.8702, p < 0.001.

*Note. b = unstandardized regression coefficient; SE = regression standard error; CI = confidence interval. The confidence intervals in this table are regression intervals, not bootstrap intervals. Indirect estimates are reported in Supplementary Table S2. FWSS = future work self salience. Perceived stress and FWSS were mean-centered before the interaction term was constructed; FWSS and its interaction with stress were included in the mediator equation, not in the outcome equation.*

# Supplementary Table S1 (continued).

## Panel B2. PROCESS Model 7 — Search for meaning

### Outcome: Search for meaning

| **Predictor** | **b** | **SE** | **t** | **p** | **95% CI** |
| --- | --- | --- | --- | --- | --- |
| Intercept | 5.0331 | 0.0940 | 53.5342 | <0.001 | [4.8487, 5.2175] |
| Perceived stress | −0.2670 | 0.0410 | −6.5096 | <0.001 | [−0.3474, −0.1865] |
| Future work self salience | 0.6074 | 0.0350 | 17.3527 | <0.001 | [0.5388, 0.6761] |
| Perceived stress × FWSS | 0.1898 | 0.0368 | 5.1518 | <0.001 | [0.1175, 0.2620] |
| Gender | −0.0028 | 0.0508 | −0.0551 | 0.9561 | [−0.1025, 0.0969] |
| Grade | −0.0240 | 0.0233 | −1.0327 | 0.3019 | [−0.0696, 0.0216] |

R² = 0.2565; F(5, 1,887) = 130.2276, p < 0.001.

### Outcome: Career adaptability

| **Predictor** | **b** | **SE** | **t** | **p** | **95% CI** |
| --- | --- | --- | --- | --- | --- |
| Intercept | 3.0081 | 0.0687 | 43.7674 | <0.001 | [2.8733, 3.1429] |
| Perceived stress | −0.3423 | 0.0187 | −18.3077 | <0.001 | [−0.3790, −0.3057] |
| Search for meaning | 0.1731 | 0.0103 | 16.7816 | <0.001 | [0.1528, 0.1933] |
| Gender | 0.0351 | 0.0248 | 1.4174 | 0.1565 | [−0.0135, 0.0837] |
| Grade | −0.0048 | 0.0113 | −0.4245 | 0.6712 | [−0.0271, 0.0174] |

R² = 0.3325; F(4, 1,888) = 235.0959, p < 0.001.

*Note. b = unstandardized regression coefficient; SE = regression standard error; CI = confidence interval. The confidence intervals in this table are regression intervals, not bootstrap intervals. Indirect estimates are reported in Supplementary Table S2. FWSS = future work self salience. Perceived stress and FWSS were mean-centered before the interaction term was constructed; FWSS and its interaction with stress were included in the mediator equation, not in the outcome equation.*

# Supplementary Table S2. Indirect associations, conditional indirect associations, and indices of moderated mediation.

| **Mediator / analysis** | **FWSS level / estimate** | **Estimate** | **BootSE** | **95% bootstrap CI** |
| --- | --- | --- | --- | --- |
| **Model 4: indirect associations** | | | | |
| Presence of meaning | Indirect association | −0.1314 | 0.0143 | [−0.1610, −0.1048] |
| Search for meaning | Indirect association | −0.1079 | 0.0127 | [−0.1343, −0.0841] |
| **Model 7: conditional indirect associations** | | | | |
| Presence of meaning | Low FWSS | −0.0832 | 0.0132 | [−0.1103, −0.0578] |
| Presence of meaning | Mean FWSS | −0.0652 | 0.0091 | [−0.0837, −0.0480] |
| Presence of meaning | High FWSS | −0.0472 | 0.0088 | [−0.0651, −0.0304] |
| Search for meaning | Low FWSS | −0.0734 | 0.0126 | [−0.1000, −0.0503] |
| Search for meaning | Mean FWSS | −0.0462 | 0.0084 | [−0.0631, −0.0308] |
| Search for meaning | High FWSS | −0.0190 | 0.0098 | [−0.0375, 0.0002] |
| **Model 7: indices of moderated mediation** | | | | |
| Presence of meaning | Index | 0.0218 | 0.0079 | [0.0067, 0.0382] |
| Search for meaning | Index | 0.0328 | 0.0090 | [0.0157, 0.0517] |

*Note. N = 1,893 for each model. Unstandardized estimates are reported. Each dimension was examined in a separate model, with gender and grade as control variables. BootSE = bootstrap standard error. All intervals are 95% percentile bootstrap intervals based on 5,000 resamples. Low and high FWSS refer to one standard deviation below and above the mean; the centered moderator values were −0.8272, 0, and 0.8272. The interval at high FWSS for search for meaning includes zero. No between-dimension contrast or summed indirect effect across the separate models was estimated.*

# Table S3. Reported measurement-model fit indices.

The values below reproduce the CFA results reported in Sections 3.3.1–3.3.4 of the revised manuscript. They are not additional model estimations.

| **Measure / model** | **χ²** | **df** | **χ²/df** | **CFI** | **TLI** | **RMSEA** | **SRMR** |
| --- | --- | --- | --- | --- | --- | --- | --- |
| PSS (14 items) | 333.426 | 75 | 4.446 | 0.979 | 0.974 | 0.043 | 0.031 |
| CAAS: correlated four-factor model | 365.701 | 246 | 1.487 | 0.995 | 0.995 | 0.016 | 0.014 |
| MLQ: correlated two-factor model | 51.936 | 34 | 1.528 | 0.998 | 0.997 | 0.017 | 0.015 |
| FWSS (5 items) | 19.271 | 4 | 4.818 | 0.997 | 0.993 | 0.045 | 0.010 |

Note. PSS = Perceived Stress Scale; CAAS = Career Adapt-Abilities Scale; MLQ = Meaning in Life Questionnaire; FWSS = future work self salience. The main manuscript identifies IBM SPSS Amos 31 as the software used for CFA and ULMC analyses. The MLQ model distinguishes presence of meaning and search for meaning.

# Table S4. Reported ULMC model comparison.

| **Model** | **χ²** | **df** | **χ²/df** | **CFI** | **TLI** | **RMSEA** | **SRMR** |
| --- | --- | --- | --- | --- | --- | --- | --- |
| Baseline measurement model | 1834.156 | 1289 | 1.423 | 0.990 | 0.989 | 0.015 | 0.019 |
| Model including a method factor | 1493.419 | 1236 | 1.208 | 0.995 | 0.995 | 0.010 | 0.016 |

Note. ULMC = unmeasured latent method factor. Relative to the baseline model, CFI increased by 0.005 and TLI by 0.006; RMSEA decreased by 0.005 and SRMR by 0.003. These comparisons do not establish that common method bias is absent. The regression estimates in the primary and sensitivity analyses are not method-bias-corrected. Values reproduce Section 4.1 of the revised manuscript.
