# Supplementary material for: The association between perceived stress and career adaptability: the mediating role of meaning in life and the moderating role of future work self salience
Source: Front Psychol. 2026 Oct 5;17:1891424. doi: 10.3389/fpsyg.2026.1891424 (PMC13677823; doi:10.3389/fpsyg.2026.1891424)
Supplement: Supplementary file 4 [file Table_2.DOCX]

# Supplementary Material 2 Questionnaire items and scoring

Chinese items and response options are reproduced from the supplied questionnaire. English translations are provided for reporting purposes and do not constitute a separately validated English instrument. The scoring notes document the scoring described in Section 3.3 of the manuscript and were not participant-facing. Item numbers are local to each measure; corresponding full-questionnaire numbers are also given.

*This supplement reports measurement content, not a facsimile or historical record of the online consent procedure. Grade in the statistical analyses refers to academic year in the questionnaire. Measure references are listed in Section 3.3 of the main manuscript.*

## Background information / 基本信息

| **中文** | **English translation** |
| --- | --- |
| 学校：[填空题] * | University: [Fill in] * |
| 您的年级：[单选题] * 大学一年级；大学二年级；大学三年级；大学四年级 | Academic year: [Select one] * First year; Second year; Third year; Fourth year |
| 您的性别：[单选题] * 男；女 | Gender: [Select one] * Male; Female |

* Indicates a required field in the supplied questionnaire. / * 表示所提供问卷中的必答项。

# Perceived Stress Scale (PSS-14) / 压力知觉量表

编号对应：本节第1–14题对应原完整问卷第1–14题。
Numbering: Local items 1–14 correspond to items 1–14 in the original full questionnaire.

## Scoring notes / 计分说明

本量表评估个体在过去一个月内的压力知觉，包含14个题项，按1（从不）至5（总是）计分。失控感维度包含第4、5、6、7、9、10、13题；紧张感维度包含第1、2、3、8、11、12、14题。第4、5、6、7、9、10、13题按“6 − 原始分”反向计分后，计算全部14题的均分。各维度得分为对应题项经必要反向处理后的均分；得分越高，表示相应的压力知觉程度越高。

The scale assesses perceived stress over the past month and contains 14 items rated from 1 (never) to 5 (always). Lack of control comprises items 4, 5, 6, 7, 9, 10, and 13; tension comprises items 1, 2, 3, 8, 11, 12, and 14. Items 4, 5, 6, 7, 9, 10, and 13 are reverse-scored as 6 minus the original response. The overall score is the mean of all 14 items after reverse scoring. Each subscale score is the mean of its constituent items after reverse scoring where applicable. Higher scores indicate higher levels of the corresponding aspect of perceived stress.

1 = 从不 / Never；2 = 偶尔 / Occasionally；3 = 有时 / Sometimes；4 = 经常 / Often；5 = 总是 / Always

| **Item** | **中文题项** | **English translation** |
| --- | --- | --- |
| 1 | 最近一个月以来，因一些无法预期的事情发生而感到心烦意乱 | Over the past month, I have felt upset because something unexpected happened. |
| 2 | 最近一个月以来，感觉无法控制自己生活中重要的事情 | Over the past month, I have felt unable to control important things in my life. |
| 3 | 最近一个月以来，感到紧张不安和压力 | Over the past month, I have felt nervous and under stress. |
| 4 | 能够成功地处理恼人的生活麻烦 | I am able to deal successfully with irritating hassles in my life. |
| 5 | 感到自己能有效地处理生活中所发生的重要改变 | I feel able to deal effectively with important changes occurring in my life. |
| 6 | 对于有能力处理自己私人的问题感到很有信心 | I feel confident in my ability to handle my personal problems. |
| 7 | 最近一个月以来，感到事情顺心如意 | Over the past month, I have felt that things were going my way. |
| 8 | 发现自己无法处理所有自己必须做的事情 | I find that I am unable to handle all the things I have to do. |
| 9 | 有办法控制生活中恼人的事情 | I have ways of controlling the irritating things in my life. |
| 10 | 常觉得自己是驾驭事情的主人 | I often feel in charge of what happens. |
| 11 | 常生气，因为很多事情的发生是超出自己所能控制的范围 | I often feel angry because many things happen beyond my control. |
| 12 | 经常想到有些事情是自己必须完成的 | I often think about things I have to accomplish. |
| 13 | 常能掌握时间的安排方式 | I am often able to organize my time. |
| 14 | 常感到困难的事情堆积如山，而自己无法克服它们 | I often feel that difficulties are piling up and that I cannot overcome them. |

# Meaning in Life Questionnaire (MLQ) / 生命意义感量表

编号对应：本节第1–10题对应原完整问卷第15–24题；反向题9对应全问卷第23题。
Numbering: Local items 1–10 correspond to original items 15–24; reverse-scored item 9 corresponds to original item 23.

## Scoring notes / 计分说明

本量表包含10个题项，采用七点计分，1表示“完全不同意”，7表示“完全同意”。存在意义感维度包含第1、4、5、6、9题；寻求意义感维度包含第2、3、7、8、10题。第9题按“8 − 原始分”反向计分后，分别计算两个五题分量表的均分。得分越高，分别表示越高的存在意义感或越强的意义寻求倾向。本研究主分析使用全部10题经反向处理后的均分，敏感性分析分别使用两个分量表的均分。

The questionnaire contains 10 items rated on a seven-point scale from 1 (completely disagree) to 7 (completely agree). Presence of meaning comprises items 1, 4, 5, 6, and 9; search for meaning comprises items 2, 3, 7, 8, and 10. Item 9 is reverse-scored as 8 minus the original response before the two five-item subscale means are calculated. Higher subscale scores indicate greater presence of meaning or a stronger search for meaning, respectively. The primary analyses in this study used the mean of all 10 items after reverse scoring; the sensitivity analyses used the two subscale means separately.

1 = 完全不同意 / Completely disagree；2 = 大部分不同意 / Mostly disagree；3 = 有些不同意 / Somewhat disagree；4 = 说不清 / Unsure；5 = 有些同意 / Somewhat agree；6 = 大部分同意 / Mostly agree；7 = 完全同意 / Completely agree

| **Item** | **中文题项** | **English translation** |
| --- | --- | --- |
| 1 | 我很了解自己的人生意义。 | I understand the meaning of my life very well. |
| 2 | 我正在寻找某种使我的生活有意义的东西。 | I am looking for something that makes my life meaningful. |
| 3 | 我总是在寻找自己的人生目标。 | I am always looking for my purpose in life. |
| 4 | 我的生活有很明确的目标感。 | My life has a very clear sense of purpose. |
| 5 | 我很清楚是什么使我的生活感觉起来是重要的东西。 | I clearly understand what makes my life feel significant. |
| 6 | 我已经发现了一个令人满意的人生目标。 | I have discovered a satisfying purpose in life. |
| 7 | 我一直在寻找某样能使我的生活感觉起来是重要的东西。 | I am constantly looking for something that makes my life feel significant. |
| 8 | 我正在寻找自己人生的目标和“使命”。 | I am searching for my purpose and “mission” in life. |
| 9 | 我的生活没有很明确的目标。 | My life does not have a very clear purpose. |
| 10 | 我正在寻找自己人生的意义。 | I am searching for meaning in my life. |

# Future Work Self Salience Scale / 未来工作自我清晰度量表

编号对应：本节第1–5题对应原完整问卷第25–29题。
Numbering: Local items 1–5 correspond to items 25–29 in the original full questionnaire.

## Scoring notes / 计分说明

本量表包含5个题项，按1（完全不符合）至5（完全符合）计分。本研究计算5个题项的均分；均分越高，表示未来工作自我清晰度越高。

The scale contains five items rated from 1 (does not describe me at all) to 5 (describes me completely). The mean of the five item scores was used in this study, with higher mean scores indicating greater future work self salience.

1 = 完全不符合 / Does not describe me at all；2 = 比较不符合 / Does not describe me very well；3 = 不确定 / Unsure；4 = 比较符合 / Describes me fairly well；5 = 完全符合 / Describes me completely

| **Item** | **中文题项** | **English translation** |
| --- | --- | --- |
| 1 | 我可以很容易地想象到未来工作中的自我形象 | I can easily imagine my future work self. |
| 2 | 这个未来的心理图像非常清楚 | The mental image of this future is very clear. |
| 3 | 这个未来对我来说是很容易想象得到的 | This future is easy for me to imagine. |
| 4 | 我非常清楚在未来工作中我想成为什么样的人 | I am very clear about the kind of person I want to become in my future work. |
| 5 | 在我的头脑中，我想要的与工作相关的未来的类型是非常清楚的 | I have a very clear picture in my mind of the work-related future I want. |

# Career Adapt-Abilities Scale (CAAS) / 生涯适应力量表

编号对应：本节第1–24题对应原完整问卷第30–53题。
Numbering: Local items 1–24 correspond to items 30–53 in the original full questionnaire.

## Scoring notes / 计分说明

本量表包含24个题项，按1（完全不符合）至5（完全符合）计分。第1–6题为生涯关注，第7–12题为生涯控制，第13–18题为生涯好奇，第19–24题为生涯自信。本研究计算全部24题的均分，四个维度的得分分别为各自6个题项的均分。得分越高，表示总体生涯适应力或相应维度的适应资源越高。

The scale contains 24 items rated from 1 (does not describe me at all) to 5 (describes me completely). Career concern comprises items 1–6, career control items 7–12, career curiosity items 13–18, and career confidence items 19–24. The overall score used in this study was the mean of all 24 items, and each subscale score was the mean of its six items. Higher scores indicate greater overall career adaptability or greater resources in the corresponding dimension.

1 = 完全不符合 / Does not describe me at all；2 = 比较不符合 / Does not describe me very well；3 = 不确定 / Unsure；4 = 比较符合 / Describes me fairly well；5 = 完全符合 / Describes me completely

| **Item** | **中文题项** | **English translation** |
| --- | --- | --- |
| 1 | 我思考过我未来的生活。 | I have thought about my future life. |
| 2 | 我明白我今天的选择决定未来的发展。 | I understand that the choices I make today determine my future development. |
| 3 | 我正在为我未来的职业生涯做准备。 | I am preparing for my future career. |
| 4 | 我关心我现在所受的教育与未来职业选择之间的关系。 | I care about the relationship between my current education and my future career choices. |
| 5 | 就如何实现我的目标，我有制定规划。 | I have made plans for how to achieve my goals. |
| 6 | 我很关心自己未来的职业生涯发展。 | I care a great deal about my future career development. |
| 7 | 我对自己未来的生涯发展保持乐观自信。 | I remain optimistic and confident about my future career development. |
| 8 | 我能够自己决定未来的职业生涯发展方向。 | I can decide for myself the direction of my future career development. |
| 9 | 我能够为自己的生涯发展负责。 | I can take responsibility for my career development. |
| 10 | 为了我未来的发展，我会一直坚持下去。 | I will persevere for the sake of my future development. |
| 11 | 我做事总是依靠自己。 | I always rely on myself to get things done. |
| 12 | 我会做那些对我未来职业发展有意义的事情。 | I will do things that are meaningful for my future career development. |

# Career Adapt-Abilities Scale (continued)

Items 13–24 / 第13–24题。Response options remain 1–5 as shown on the preceding page.

| **Item** | **中文题项** | **English translation** |
| --- | --- | --- |
| 13 | 我喜欢探索周围的事物。 | I like to explore the things around me. |
| 14 | 为了更快的成长，我会努力寻找更多的机会。 | I strive to find more opportunities so that I can develop more quickly. |
| 15 | 在做一些重要选择之前，我会先查找相关的资料。 | Before making important choices, I first look for relevant information. |
| 16 | 我会观察、学习生活中人们处事的不同方式。 | I observe and learn from the different ways people handle situations in everyday life. |
| 17 | 对于职业生涯方面遇到的问题，我会深入探究。 | I look into the career-related problems I encounter in depth. |
| 18 | 我会留意生活中的一切机会。 | I look out for every opportunity in life. |
| 19 | 我相信自己能有效的完成自己的工作/任务。 | I believe I can complete my work/tasks effectively. |
| 20 | 我关心自己能否把事情做好。 | I care about whether I can do things well. |
| 21 | 我会尝试新的技能来提高自己的能力。 | I try out new skills to improve my abilities. |
| 22 | 我相信自己的能力会得到不断提升。 | I believe my abilities will continue to improve. |
| 23 | 我相信自己能够克服生涯发展中的障碍，获得成功。 | I believe I can overcome obstacles in my career development and achieve success. |
| 24 | 我相信自己能够解决生涯发展中遇到的问题。 | I believe I can solve the problems I encounter in my career development. |
